# Comparing age of obesity onset, concerns about weight and body shape, and quality of life in individuals seeking bariatric surgery

**DOI:** 10.55730/1300-0144.6344

**Published:** 2026-04-30

**Authors:** Vildan ÇAKIR KARDEŞ, Özge SARAÇLI, Nuray ATASOY, Levent ATİK, Sibel KAHRAMAN GİRGEÇ

**Affiliations:** 1 Department of Psychiatry, Faculty of Medicine, Zonguldak Bülent Ecevit University, Zonguldak, Turkiye; 2 Department of Psychiatry, Abdulkadir Yüksel State Hospital, Gaziantep, Turkiye

**Keywords:** Concerns about body shape, quality of life, bariatric surgery, childhood- or adolescent-onset obesity, adult-onset obesity

## Abstract

**Background/aim:**

This study explored the relationship between concerns about weight and body shape and quality of life in bariatric surgery candidates with morbid obesity regarding the onset of the weight problem in childhood, adolescence, or adulthood.

**Materials and methods:**

We included 453 patients who applied to our center for preoperative bariatric surgery assessment between January 2010 and April 2019. We reviewed the sociodemographic information, psychiatric diagnoses, and results of the Body Shape Questionnaire-34, Quality of Life Scale, Beck Depression Inventory, and Beck Anxiety Inventory of all patients.

**Results:**

Concerns about weight and body shape were increased in women (p < 0.001), unemployed patients (p = 0.001), those with a comorbid psychiatric disorder (p < 0.001), and those with childhood- or adolescent-onset of weight problems (r = −0.170; p < 0.001). Concerns about weight and body shape had a relationship with the vitality (r = −0.434), mental health (r = −0.464), social functioning (r = −0.418), pain (r = −0.307), and general health (r = −0.352) subscale scores of quality of life. Physical functioning was higher (p = 0.037) in patients with childhood- or adolescent-onset obesity compared to patients with adult-onset obesity, while mental health was higher (p = 0.001) in the latter group. Individuals with childhood- or adolescent-onset obesity inquired about bariatric surgery options at an earlier age (p < 0.001).

**Conclusion:**

While performing routine psychiatric evaluations before bariatric surgery, it is important to consider parameters such as the patient’s physical functioning, level of concerns about the body, and age of onset of obesity.

## 1. Introduction

Morbid obesity is the most severe condition of obesity and can lead to both medical and social problems. These patients frequently present with psychiatric disorders as well as medical conditions. They are adversely affected by accompanying psychiatric disorders, medical conditions, failed weight-loss attempts, and social pressures to lose weight. Quality of life (QoL) also decreases with the high anxiety levels triggered by monitoring one’s body and engaging in avoidance behaviors. Studies have suggested a decrease in QoL with increased body mass index (BMI) levels [1]. Low QoL was reported as the major reason for preferring the bariatric surgery method among patients with morbid obesity [2,3].

BMI has had a significant impact on body dissatisfaction in studies about body perception in people with obesity [4], and patients with obesity were found to be more dissatisfied with their bodies than nonobese individuals [5]. More than 80% of women had negative body image in a study conducted using the Body Shape Questionnaire (BSQ) with women with obesity who were undergoing therapy due to their negative body perceptions [6]. Body dissatisfaction severity in women with obesity was more associated with perceived body weight than actual body weight [5]. Studies indicate that many factors adversely affect body perception in individuals with obesity. One study reported that slightly overweight women had the same attitudes toward their physical appearances as individuals with obesity [7].

In this study, we aimed to assess the impact of some specific factors on the QoL and body image of candidate patients for bariatric surgery, including demographic variables, anxiety and depression levels, BMI, timing of obesity onset, factors associated with obesity (exercise, diet, etc.), and history of obesity and psychiatric disorders in the families of these candidate patients for bariatric surgery. The study’s hypotheses were as follows:

H1: It is hypothesized that being female and having a psychiatric comorbidity are significant predictors of elevated concerns about body shape and weight in patients awaiting bariatric surgery.
H2: There will be a significant difference in body-image concerns based on the timing of obesity onset, with early-onset individuals exhibiting higher levels of weight-related distress than their adult-onset counterparts.
H3: Early-onset obesity is associated with an earlier age of seeking bariatric surgery.
H4: Surgery candidates with adult-onset obesity will demonstrate higher physical function scores and fewer obesity-related comorbidities than candidates with childhood- or adolescent-onset obesity.

## 2. Materials and methods

In our center, we carry out routine medical examinations of patients who are admitted to our clinic with the hope of undergoing bariatric surgery. These patients are evaluated in a multidisciplinary manner by endocrinologists, general surgeons, dietitians, psychiatrists, cardiologists, pulmonologists, physical medicine practitioners, and rehabilitation physicians. During the preoperative psychological evaluation, the patients are examined regarding their understanding of the surgical procedure, decision-making, and postoperative adjustment and management skills. Current psychiatric disorders are documented at the baseline interview by trained clinicians (master’s level or higher) using the Structured Clinical Interview for DSM-IV-TR Axis I Disorders [8]. Data on eating disorders that may play a role in the etiology of obesity were excluded from the analysis of this study due to missing data in the records.

We reviewed the files, including sociodemographic information, psychiatric diagnoses, and the results of the BSQ-34, Short Form-36 Quality of Life Scale (SF-36), Beck Anxiety Inventory (BAI), and Beck Depression Inventory (BDI) for all patients who were admitted to the Psychiatry Clinic of the Bülent Ecevit University Faculty of Medicine for preoperative assessment for bariatric surgery between January 2010 and April 2019. The study was approved by the Bülent Ecevit University Clinical Studies Ethics Committee on 22 May 2019 with approval number 2019/08.

### 2.1. Sociodemographic information

We reviewed demographic information such as age, sex, marital and educational status, occupation, alcohol consumption, smoking status, and other medical conditions of the patients. Data were collected regarding the patients’ family history of psychiatric disorders and obesity, diet history, exercise history, daily screen time, and the onset and duration of the weight problem. To define age at the onset of obesity, we used the categories of “childhood-adolescent onset (5–17 years)” and “adult onset (18+ years).” We estimated the duration of obesity by asking the participants.

### 2.2. Body mass index (BMI)

BMI was calculated as weight (kg) divided by height (m^2^).^1^

### 2.3. Short Form-36 (SF-36)

We used the Turkish version of the SF-36, which consists of the 8 subscales of physical functioning, physical role limitations, emotional role limitations, vitality, mental health, social functioning, pain, and general health perceptions. Every subscale is scored in the range of 0 to 100. Higher scores indicate better health and higher levels of functioning [9].

### 2.4. Beck Depression Inventory (BDI)

Developed by Beck et al., this self-report scale consists of 21 questions to assess the severity of depression symptoms [10]. It is a 4-point Likert-type scale with scores ranging from 0 to 63. Hisli adapted the scale to Turkish [11].

### 2.5. Beck Anxiety Inventory (BAI)

Developed by Beck et al., this self-assessment scale consists of 21 questions [12]. The total score ranges from 0 to 23, with higher scores reflecting a greater level of anxiety severity. Ulusoy et al. carried out the Turkish validity and reliability study of the scale [13].

### 2.6. Body Shape Questionnaire (BSQ)

Data on body image was obtained using the BSQ, which measures concerns about the individual’s weight and body shape. Cooper et al. developed the scale in 1987 and determined its reliability [14]. Akdemir et al. determined the validity and reliability of the Turkish version of the scale [15]. The BSQ is a one-dimensional scale containing 34 items scored with 1–6 points (1: “never,” 2: “rarely,” 3: “sometimes,” 4: “often,” 5: “very often,” and 6: “always”). A score of <80 indicates no anxiety while increasing scores indicate higher levels of engagement with body image and weight and greater anxiety. The Cronbach alpha internal reliability and consistency coefficient was 0.88 for the scale.

### 2.7. Statistics

SPSS 18.0 (SPSS Inc., Chicago, IL, USA) was used for statistical evaluation. The Shapiro–Wilk test was used to test whether the numerical variables were normally distributed. Numerical variables were expressed as mean ± standard deviation and median (minimum–maximum), while categorical variables were presented as frequencies and percentages. The chi-square test was used for the analysis of differences and the relationships between groups regarding categorical variables. When the data met the assumptions of the parametric test in the comparison of two groups regarding numerical variables, we used hypothesis testing of the difference between the means of the two groups, and we used the Mann–Whitney U test when those assumptions were not met. We used Pearson correlation analysis when our data met the assumptions of the parametric test of the linear relationship between two numerical variables and we used Spearman correlation analysis when those assumptions were not met. The confidence interval was 95% and values of p < 0.05 were considered significant.

## 3. Results

Of all bariatric surgery candidates in our hospital, 320 (70.6%) were women and 133 (29.4%) were men. The mean age was 39.40 ± 10.80 (range: 16–67). According to the sociodemographic evaluations, 41.7% of the patients were primary school graduates, 72% were married, and 49% were housewives or unemployed. Table 1 presents the sociodemographic information and features of the medical and family history of the analyzed bariatric surgery candidates.

The mean BMI was 47.30 ± 7.20 (range: 35.60–78.07), including classes II and III of obesity. The mean duration of the weight problem was 17.99 ± 9.70 years (range: 2–57). Adult and childhood-adolescent onset had occurred for 53.2%, and 46.8% of the patients, respectively. While 69.8% reported that they had tried diets that did not work, 10.2% had a history of laxative use. Half of the patients indicated that they did not do any exercise, 18.3% reported exercising regularly, and 31.1% reported exercising irregularly. Furthermore, 28.7% had a psychiatric disorder, the most frequent of which were major depression, anxiety disorder, and adjustment disorder. We did not observe any suicidal thoughts in these patients during our examinations; however, 3.1% of the patients had a history of attempted suicide. The distributions of psychiatric diagnoses and scores of the patients are shown in Table 2.

The relationships between BSQ scores and sociodemographic and descriptive characteristics are shown in Table 3. The mean BSQ score of women was significantly higher than that of men (p < 0.001). The BSQ scores of the unemployed/housewife group were significantly higher than those of the employed group (p = 0.001). The mean BSQ score of the group with psychiatric disorders was significantly higher than that of the group without any psychiatric disorders (p < 0.001).

Table 4 presents the results of correlation analysis performed for the numerical variables of BSQ, SF-36, BDI, and BAI scores and age, BMI, and duration of the weight problem. Significant negative correlations were found between age and duration of the weight problem and BSQ scores (respectively r = −0.137, p = 0.004; r = −0.170, p < 0.001).

There were also negative correlations between BSQ scores and all SF-36 subscale scores (Table 4). However, there was no significant relationship between BMI and BSQ scores (r = 0.064, p > 0.05). When comparing the demographic characteristics of the participants based on the onset of obesity, statistically significant differences were found between the groups in terms of marital status (p < 0.001), occupation (p = 0.001), presence of physical disorder (p = 0.027), and daily exercise duration (p = 0.005). Among those whose obesity began in adulthood, the rates of being married and having a physical disorder were higher. Bonferroni-corrected post hoc comparisons revealed significant differences according to the timing of obesity onset. Specifically, the proportion of students was higher among participants with childhood-adolescent onset of obesity compared to those with adult-onset obesity (p < 0.05). Distinct patterns were also observed in physical activity: the group with childhood-adolescent onset exhibited a significantly higher frequency of daily exercise lasting 30–60 min, whereas the group with adult onset was significantly more likely to report no exercise at all (p < 0.05). In contrast, no statistically significant differences were identified between the groups for sex, psychiatric diagnosis, laxative use, or dietary habits (p > 0.05).

Differences in scale scores and numerical data between the groups according to the time of onset of the weight problem are shown in Table 5. Individuals with childhood- or adolescent-onset sought bariatric surgery options at earlier ages (p < 0.001). Predictably, a relationship was found between earlier onset of obesity and longer duration of obesity. No difference was found between the two groups regarding BMI values (p = 0.631).

Individuals with early-onset obesity (childhood or adolescent onset) demonstrated significantly higher BSQ scores than their adult-onset counterparts. The independent variables in the model explained approximately 28.5% of the variance in BSQ scores. It was determined that a 1-unit increase in BDI score corresponded to a 1.528-point increase in BSQ score, a 1-unit increase in age reduced the BSQ score by 0.367 points, being female increased the BSQ score by 14.541 points, and the onset of obesity occurring during childhood or adolescence increased the BSQ score by 8.128 points (Table 6).

## 4. Discussion

Although morbid obesity is widely known to reduce individual QoL, it is controversial whether factors such as physical health, mental health, self-confidence, and relationships with others have any impact on this reduction [2,16,17]. The present study found that weight and body shape concerns in patients with morbid obesity adversely affected their QoL.

In some studies examining bariatric surgery candidates, the rate of female patients was 71%–83%, the mean age ranged from 38 to 46 years, and the mean BMI was 49–52 [18,19]. Similarly, in our study, 70.6% of the patients were women, the mean age was 39.40 ± 10.80, and the mean BMI was 47.30 ± 7.20. Another previous study had similar findings, showing that patients seeking bariatric surgery were mostly women [20].

Similar to our study, the most common preoperative psychiatric diagnoses were reported in the literature as depression and anxiety disorder among bariatric surgery candidates [21–23]. Researchers have demonstrated that candidates for bariatric surgery have a higher prevalence of psychopathologies compared to other people with obesity in the general population. The psychiatric diagnosis rate in our research group (28.7%) aligns with the range reported in the literature (20% to 60%) [18,24–26].

There are limited studies in the literature investigating the prevalence of a history of suicide attempts in patients seeking bariatric surgery. In a study by Sansone et al., 121 bariatric surgery patients were evaluated and the prevalence of suicide attempts and self-harming behaviors were examined, and 9.1% of the participants were found to have a history of suicide attempts [27]. Another study reported that 11.2% of candidates for bariatric surgery had attempted suicide once or more than once in their lifetimes (n = 1020) [28]. In our study, we did not identify any patient with suicidal thoughts during our examinations; however, 3.1% of the patients had a history of attempted suicide. This rate is lower than the rates found in the literature. Previous suicide attempts are the strongest indicator of the likelihood of future attempts [29]. Current suicidal thoughts and recent (within the past year) suicide attempts are said to be contraindications for bariatric surgery [30]. In this context, it is critical to ask about suicide attempt history and thoughts in patients who consult with the hope of undergoing bariatric surgery.

Although the prevalence of constipation in individuals with obesity has been reported at rates as high as 25.3% in the literature [31], our study found that the rate of laxative use remained at 10%. This discrepancy may stem from our study assessing only the use of pharmacological treatments. Another study reported a laxative usage rate of 32% among participants with BMI of 30 kg/m^2^ or higher [32], which is significantly higher than the 10% usage rate observed in our study. The tendency of individuals to rely on traditional methods (herbal teas, functional foods, etc.) to manage constipation symptoms or the fact that constipation symptoms may not be severe enough to warrant medication use may explain the low medication usage rate observed in this study.

Studies on body dissatisfaction levels among patients with obesity showed that women were more dissatisfied with their bodies than men. Overweight men wished to be slimmer than normal or underweight men, and women still wanted to lose weight even when they were of normal weight or underweight [33,34]. Other studies on sex differences reported that body dissatisfaction was lower in men than women [35]. In our study, we also noted higher concern about body shape among women compared to men. The reasons for this finding may include biological factors and aesthetic concerns arising from societally imposed gender roles.

Many studies have reported higher rates of body-image dissatisfaction in women with obesity compared to those in the normal weight range; however, some studies indicated no relation between body-image dissatisfaction and BMI in obese and overweight women [5,36]. Deviating from the majority of the literature, the expected correlation between BMI and body-image dissatisfaction was not observed in our study, consistent with only a limited number of prior investigations [37,38]. This finding may have resulted from the mean BSQ score being much higher than 80 (133.35 ± 37.3), which indicates a high level of anxiety regarding body image across the entire study group. This divergence might be explained by a “threshold effect,” suggesting that once body weight reaches a certain level, body-image dissatisfaction plateaus rather than continuing to increase alongside BMI [38]. Our findings are consistent with the psychological theory that body-image dissatisfaction stems not so much from the objective reality of a person’s physical dimensions as from subjective inner feelings of distress, such as shame and anxiety.

In our study, BSQ scores were notably higher in unemployed individuals and housewives compared to those who were working or retired. Based on this finding, we suggest that having a job affects a person’s self-perception positively. In studies carried out in developed countries, the risk of being overweight and obese was emphasized to decrease with the possibility of healthy nutrition intake due to high income [39]; however, we found little data on the effect of having a job on self-perception in patients with obesity. In addition, when we used advanced analytical techniques, we found that employment status had no significant effect on predicting BSQ scores. Further research is needed on the impact of employment status on body perception.

As BMI values increased, the physical functioning, physical role limitations, emotional role limitations, vitality, mental health, social functioning, pain, and general health subscale scores of QoL decreased, and this finding was compatible with findings in the literature. A weak correlation was found between BMI and SF-36 subscale scores. Furthermore, a moderate negative correlation was found between BSQ scores and the vitality, mental health, social functioning, pain, and general health subscale scores of QoL. According to these findings, body perception affects QoL more than BMI.

In this study, a moderate positive correlation was found between BSQ body perception scores and both BDI and BAI scores. Additionally, the mean BSQ score of patients with a psychiatric disorder was significantly higher than that of patients without a psychiatric disorder (p < 0.001; Table 4). These findings suggest that concerns about weight and body shape, the presence of a psychiatric disorder, and levels of anxiety and depression may be interrelated.

Consistent with the literature, we found that patients with childhood- or adolescent-onset obesity experienced higher levels of body dissatisfaction compared to those with adult-onset obesity [40]. Hill and Williams showed that most women with obesity were likely to have been overweight since childhood; moreover, they had poorer body image [41]. However, Hill and Williams did not analyze the association between age of onset and body dissatisfaction; instead, they examined the relationship between early onset of obesity and higher levels of body dissatisfaction in adulthood. In the literature, childhood-onset obesity is found to be related to weight-based stigmatization in childhood and, accordingly, it is also found to be more closely related to body dissatisfaction and negative body image in adulthood [40,42]. The present study has shown that concerns about body shape decrease with increased age and longer duration of being overweight. Additionally, in our study, no difference was found in terms of BMI scores between individuals with childhood- or adolescent-onset obesity. Based on these results, individuals’ self-assessments during the development of their self-concept appear to be more important than the mere fact of being overweight or the duration of being overweight.

Our study found that individuals with childhood- or adolescent-onset weight problems sought bariatric surgery at an earlier age. This finding correlates with previous studies claiming that early-onset obesity increases a patient’s possibility of desiring bariatric surgery [43,44]. In the present study, we found that individuals whose weight issues began in childhood or adolescence had fewer physical disorders, demonstrated better daily activity levels, and had higher physical function scores in terms of QoL. However, they sought bariatric surgery at younger ages compared to those whose weight problems had started in adulthood. In patients whose obesity began in childhood, the decision to undergo bariatric surgery at a younger age may stem from more serious concerns about weight and mental health issues; this highlights the particularly adverse psychological effects of obesity during early developmental stages compared to cases that begin in adulthood. Additionally, having experienced the failure of countless diets and exercise programs tried since childhood may convince these individuals to opt for surgical treatment, viewed as the “gold standard,” at an earlier age. Despite being chronologically younger when they pursue bariatric surgery, these patients have actually lived with obesity for a much longer period than those who develop it as adults. When measured from the start of their condition, their timeline to surgical evaluation suggests a significant delay in treatment rather than a truly early or proactive medical intervention.

In our study, bariatric surgery patients with childhood- or adolescent-onset obesity reported a lower prevalence of physical disorders compared to those with adult-onset obesity, despite having been obese for a longer duration of time. Our finding correlates with another study reporting that patients with early-onset obesity had a lower prevalence of metabolic disorders [44]. This suggests that the developmental timing of obesity onset is a critical factor in shaping the metabolic health profile of bariatric surgery candidates. Our study further showed that the age of onset of obesity is a significant factor that should be considered in evaluating patients before bariatric surgery. Contrary to the view that long-term obesity is associated with more physical disorders, our findings support the idea that the age of onset of obesity is more important than the duration of obesity concerning physical disorders associated with obesity.

Subscale scores for QoL physical functioning were higher in patients with childhood- or adolescent-onset obesity compared to patients with adult-onset obesity, but mental health was found to be higher in the latter group. As far as we know, there are no previous studies in the literature exploring the relationship between the onset of obesity and QoL. Individuals with childhood- or adolescent-onset obesity may have better body adaptation in terms of the musculoskeletal system or movement skills, and so they might have less difficulty in physical functioning than those with adult-onset obesity. This finding is supported by the groups’ exercise durations: individuals with childhood- or adolescent-onset obesity reported exercising daily for at least 30 min, but those with adult-onset obesity were found to do no exercise or exercise for only a short period of time. However, adolescents with obesity were previously found to be at critical risk of increased psychosocial impairment and weak developmental adaptation [45]. Thus, it is possible to claim that the mental health of individuals with obesity is negatively affected during times when they give much importance to their appearances and have a less-developed sense of self. Recent research has shown that childhood weight stigma harms psychological functioning in early childhood with the effects remaining visible in adulthood [46]. Considering these findings, the differences between the two groups of the present study in QoL subdimensions suggest that the superior physical functioning observed in the early-onset group likely stems from their younger ages or long-term functional adaptation. Conversely, their diminished mental health and heightened body-image anxiety may be attributed to the cumulative psychological strain associated with living with obesity since childhood or adolescence.

To our knowledge, this is the first study to evaluate body-image concerns and QoL in the context of age of onset of obesity in individuals seeking bariatric surgery. The study has several limitations to be acknowledged. First, the design of the study was cross-sectional, and so it does not present any cause-and-effect relationships. Second, the data were collected from bariatric surgery candidates in a tertiary hospital and the findings cannot be generalized to all individuals with morbid obesity. These patients completed the administered scales before undergoing bariatric surgery operations, and so they might have overstated their situations. The use of self-report scales for measuring QoL and body shape perception is another limitation of this study. Furthermore, patients with eating disorders were not excluded from the study and data on how many of the participants ultimately underwent bariatric surgery were not available.

This study has shown that concerns about weight and body shape were increased in women, in unemployed individuals, in the presence of psychiatric comorbidities, and when the onset of the weight problem occurred in childhood or adolescence; however, such concerns decreased with age and the duration of the weight problem. As concerns about weight and body shape increased, all QoL subdimension scores worsened.

Furthermore, we found that individuals whose weight issues began in childhood or adolescence experienced fewer physical disorders, demonstrated better daily activity levels, and had higher physical function scores in terms of QoL subdimensions. However, they sought bariatric surgery at a younger age compared to those whose weight problems had started in adulthood. The tendency to seek earlier bariatric surgery may be related to individuals with childhood- or adolescent-onset obesity having stronger concerns about their weight and body shape, as well as facing more mental health issues compared to those whose obesity started in adulthood. The mental health of individuals with childhood- or adolescent-onset obesity was found to be more negatively affected compared to individuals with adult-onset obesity, and this finding is remarkable. Additionally, concerns about weight and body shape were found to have relationships with BDI and BAI scores and the vitality, mental health, social functioning, pain, and general health subdimensions of QoL. In addition to considering these findings in psychiatric evaluations before bariatric surgery, protective measures to prevent obesity should be generally applied in both health and social fields to prevent these effects from starting in childhood.

## Figures and Tables

**Table 1:** Sociodemographic characteristics and medical and family histories of the study group.

		Number (n)	Percent (%)
	Total	453	100
Sex	Female	320	70.6
	Male	133	29.4
Marital status	Married	326	72.0
	Single	110	24.3
	Widow/divorced	17	3.8
Educational status	Primary school	189	41.7
	Secondary school	64	14.1
	High school	110	24.3
	University and beyond	90	19.9
Occupation	Housewife/unemployed	222	49.0
	Employed	179	39.5
	Retired	26	5.7
	Student	26	5.7
Smoking	Nonsmoker	253	55.8
	Ex-smoker	29	6.4
	Smoking	171	37.7
Alcohol	No consumption	392	86.5
	Previous consumption	10	2.2
	Current consumption	51	11.3
Comorbid physical disorder	Yes	198	43.7
	No	255	56.3
Comorbid endocrine disorder	Yes	128	28.3
	No	325	71.7
Family history of obesity	Yes	274	60.5
	No	179	39.5
Family history of psychiatric disorder	Yes	44	9.7
	No	409	90.3

**Table 2:** Psychiatric diagnoses and scale scores.

		Number (n)	Percent (%)

History of any suicide attempt	Yes	14	3.1
	No	439	96.9

No psychiatric diagnosis		323	71.3

Major depression		66	14.6

Anxiety disorders (panic disorder, general anxiety disorder, social anxiety disorder, specific phobia, posttraumatic stress disorder)		35	7.7

Adjustment disorder		22	4.9

Obsessive compulsive disorder		3	0.7

Schizophrenia		3	0.7

Alcohol abuse syndrome		1	0.2

Total		453	100

	Mean ± SD	Median	Range

Body Shape Questionnaire	133.35 ± 37.3	141	36–203

Beck Depression Inventory	14.41 ± 9.9	13	0–47

Beck Anxiety Inventory	11.55 ± 10.6	8	0–51

Physical functioning	48.05 ± 23.5	50	0–100

Physical role limitations	42.28 ± 38.4	25	0–100

Emotional role limitations	50.63 ± 29.9	33	0–100

Vitality	44.28 ± 21.1	45	0–95

Mental health	59.23 ± 20.2	60	0–100

Social functioning	54.53 ± 28.1	50	0–100

Pain	58.17 ± 31.9	57	0–100

General health perception	41.14 ± 23.2	40	0–100

**Table 3:** Relationships between Body Shape Questionnaire (BSQ) scores and sociodemographic and descriptive data.

		BSQ		p
		Mean ± SD	Median (range)	
Sex	Female	140.42 ± 34.5	148 (36–203)	**<0.001**
	Male	116.65 ± 38.2	119 (38–201)	
Marital status	Married	132.68 ± 38.5	142 (36–201)	0.308
	Single	135.19 ± 33.2	141 (55–203)	
	Widow/divorced	134.25 ± 40.5	134 (56–191)	
Educational status	Primary school	135.01 ± 36.7	143 (36–203)	0.716
	Secondary school	133.95 ± 38.9	144 (55–200)	
	High school	132.02 ± 38.6	137 (38–201)	
	University and beyond	131.12 ± 35.9	140 (50–192)	
Occupation	Housewife/unemployed	140.58 ± 33.9	148 (36–203)	**0.001**
	Employed	124.86 ± 40.9	132 (38–201)	
	Retired	125.35 ± 34.7	123 (71–179)	
	Student	138.46 ± 26.7	144 (79–180)	
Laxative use	Yes	142.91 ± 30.8	150 (80–190)	0.101
	No	132.28 ± 37.7	140 (36–203)	
Dieting	Yes	134.46 ± 36.1	141 (36–203)	0.608
	No	130.79 ± 39.8	142 (38–188)	
Exercise	No	131.03 ± 37.7	135 (38–201)	0.381
	Sometimes	135.12 ± 36.9	143 (38–203)	
	Regularly	136.67 ± 36.3	147 (36–200)	
Physical disorder	Yes	129.73 ± 40.1	136 (38–201)	0.145
	No	136.12 ± 34.7	143 (36–203)	
Endocrine disorder	Yes	131.27 ± 38.8	141 (38–201)	0.544
	No	134.15 ± 36.6	141 (36–203)	
Family history of obesity	Yes	134.37 ± 35.5	141 (38–203)	0.540
	No	131.75 ± 39.8	141 (36–201)	
Psychiatric disorder diagnoses	Yes	148.84 ± 30.6	156 (67–203)	**<0.001**
	No	127.06 ± 37.9	135 (36–201)	
History of any suicide attempt	Yes	156.36 ± 36.3	169 (72–200)	0.139
	No	132.60 ± 37.1	141 (36–203)	

Significant results are shown in bold.

**Table 4:** Results of the correlation analysis between scale scores of patients and numerical variables.

	Age	BMI	Duration of weight problem (years)	BDI	BAI	Physical functioning	Role-physical	Role-emotional	Vitality	Mental health	Social functioning	Pain	General health
r	r	r	r	r	r	r	r	r	r	r	r	r
Age	**–**												
BMI	**0.176** **	–											
Duration of weight problem (years)	**0.447** **	0.085	–										
BDI	0.023	**0.239** **	−0.039	**–**									
BAI	0.043	**0.181** **	−0.019	**0.632** **	**–**								
Physical functioning	**−0.279** **	**−0.259** **	−0.071	**−0.271** **	**−0.254** **	**–**							
Role-physical	**−0.147** **	**−0.197** **	0.008	**−0.350** **	**−0.256** **	**0.436** **	**–**						
Role-emotional	−0.034	**−0.124** **	0.060	**−0.258** **	**−0.199** **	**0.191** **	**0.484** **	**–**					
Vitality	−0.069	**−0.112** *	0.070	**−0.562** **	**−0.427** **	**0.368** **	**0.421** **	**0.275** **	**–**				
Mental health	0.063	**−0.093** *	**0.122** **	**−0.582** **	**−0.428** **	**0.267** **	**0.280** **	**0.231** **	**0.661** **	–			
Social functioning	**−0.115** *	**−0.174** **	0.001	**−0.440** **	**−0.318** **	**0.493** **	**0.498** **	**0.342** **	**0.592** **	**0.488** **	–		
Pain	**−0.186** **	**−0.231** **	−0.011	**−0.483** **	**−0.490** **	**0.383** **	**0.472** **	**0.262** **	**0.440** **	**0.364** **	**0.468** **	–	
General health	−0.067	**−0.216** **	0.076	**−0.472** **	**−0.414** **	**0.456** **	**0.433** **	**0.269** **	**0.596** **	**0.469** **	**0.545** **	**0.539** **	–
BSQ	**−0.137** **	0.064	**−0.170** **	**0.469** **	**0.326** **	**−0.267** **	**−0.263** **	**−0.174** **	**−0.434** **	**−0.464** **	**−0.418** **	**−0.307** **	**−0.352** **

BMI: Body mass index; BDI: Beck Depression Inventory; BAI: Beck Anxiety Inventory; BSQ: Body Shape Questionnaire;

* p < 0.05;

** p < 0.001; significant results are shown in bold.

**Table 5:** Differences between groups according to the time of onset of the weight problem.

		Onset of weight problem		p

		Childhood-adolescent onset	Adult onset	

Age	Median (range)	33.50 (16–65)	43.00 (21–67)	**<0.001** **
	Mean ± SD	34.29 ± 9.77	43.90 ± 9.61	

BMI	Median (range)	45.98 (35.92–81.58)	45.32 (36.00–92.27)	0.631
	Mean ± SD	47.55 ± 8.01	47.80 ± 7.88	

Duration of obesity (years)	Median (range)	20.00 (5–75)	13.00 (2–40)	**<0.001** **
	Mean ± SD	22.86 ± 9.82	13.70 ± 7.27	

BDI	Median (range)	14.00 (0–47)	13.00 (0–43)	0.142
	Mean ± SD	15.03 ± 9.73	13.86 ± 10.01	

BAI	Median (range)	9.00 (0–51)	8.00 (0–51)	0.203
	Mean ± SD	12.17 ± 10.81	11.00 ± 10.49	

BSQ	Median (range)	146.50 (42–201)	135.00 (36–203)	**<0.001** **
	Mean ± SD	140.96 ± 32.17	126.72 ± 40.07	

Physical functioning	Median (range)	50.00 (0–100)	45.00 (0–100)	**0.037** *
	Mean ± SD	50.38 ± 22.76	46.04 ± 24.14	

Role-physical	Median (range)	25.00 (0–100)	25.00 (0–100)	0.724
	Mean ± SD	41.27 ± 37.79	42.95 ± 38.89	

Role-emotional	Median (range)	33.33 (0–100)	66.67 (0–100)	0.309
	Mean ± SD	49.06 ± 28.92	51.73 ± 30.55	

Vitality	Median (range)	40.00 (0–95)	45.00 (0–95)	0.178
	Mean ± SD	43.11 ± 19.53	45.62 ± 22.33	

Mental health	Median (range)	56.00 (0–100)	64.00 (0–100)	**0.001** **
	Mean ± SD	56.43 ± 19.45	61.98 ± 20.54	

Social functioning	Median (range)	50.00 (0–100)	50.00 (0–100)	0.890
	Mean ± SD	54.54 ± 28.00	54.51 ± 28.17	

Pain	Median (range)	65.00 (0–100)	57.50 (0–100)	0.890
	Mean ± SD	58.28 ± 31.41	58.03 ± 32.02	

General health	Median (range)	37.50 (0–100)	45.00 (0–100)	0.151
	Mean ± SD	39.62 ± 22.10	42.57 ± 23.94	

BMI: Body Mass Index; BDI: Beck Depression Inventory; BAI: Beck Anxiety Inventory; BSQ: Body Shape Questionnaire;

* p<0.05;

** p<0.001; significant results are shown in bold.

**Table 6:** Results of multiple linear regression analysis for predictors of BSQ scores.

Variables	B	Std. error	β	t	p

Constant	120.326	12.572	–	9.571	**<0.001**

BDI	1.528	0.203	0.406	7.525	**<0.001**

Sex					
Men (ref.)	–	–	–	–	–
Women	14.541	4.352	0.179	3.341	**0.001**

**Onset of weight problem**					
Adult onset (ref.)	–	–	–	–	–
Childhood-adolescent onset	8.126	3.416	0.109	2.379	**0.018**

Age	−0.367	0.181	−0.107	−2.023	**0.044**

BMI	−0.203	0.201	−0.043	−1.007	0.315

BAI	0.113	0.184	0.032	0.613	0.540

Occupation					
Housewife/unemployed (ref.)	–	–	–	–	–
Retired	7.139	7.712	0.045	0.926	0.355
Student	−3.495	7.349	−0.022	−0.476	0.635
Employed	−1.048	4.141	−0.014	−0.253	0.800

R^2^ = 0.285, F(9, 437) = 19.374, p < 0.001. BMI: Body mass index; BDI: Beck Depression Inventory; BAI: Beck Anxiety Inventory; BSQ: Body Shape Questionnaire; significant results are shown in bold.

## Data Availability

The datasets generated in this study are not publicly available due to privacy regulations and the terms of participant informed consent. However, anonymized data are available from the corresponding author on reasonable request.

## Footnotes

1 World Health Organization (2018). Obesity and Overweight [online]. Website http://www.who.int/mediacentre/factsheets/fs311/en/ [accessed 21 March 2021].
